# ESR concept paper on value-based radiology

**DOI:** 10.1007/s13244-017-0566-1

**Published:** 2017-08-30

**Authors:** European Society of Radiology (ESR)

**Affiliations:** Neutorgasse 9/2, 1010 Vienna, Austria

**Keywords:** Value-based healthcare, Value-based radiology, Health economics, Performance metrics, Patient outcomes

## Abstract

The European Society of Radiology (ESR) established a Working Group on Value-Based Imaging (VBI WG) in August 2016 in response to developments in European healthcare systems in general, and the trend within radiology to move from volume- to value-based practice in particular. The value-based healthcare (VBH) concept defines “value” as health outcomes achieved for patients relative to the costs of achieving them. Within this framework, value measurements start at the beginning of therapy; the whole diagnostic process is disregarded, and is considered only if it is the cause of errors or complications. Making the case for a new, multidisciplinary organisation of healthcare delivery centred on the patient, this paper establishes the diagnosis of disease as a first outcome in the interrelated activities of the healthcare chain. Metrics are proposed for measuring the quality of radiologists’ diagnoses and the various ways in which radiologists provide value to patients, other medical specialists and healthcare systems at large. The ESR strongly believes value-based radiology (VBR) is a necessary complement to existing VBH concepts. The Society is determined to establish a holistic VBR programme to help European radiologists deal with changes in the evolution from volume- to value-based evaluation of radiological activities.

*Main Messages*

*• Value-based healthcare defines value as patient’s outcome over costs.*

*• The VBH framework disregards the diagnosis as an outcome.*

*• VBH considers diagnosis only if wrong or a cause of complications.*

*• A correct diagnosis is the first outcome that matters to patients.*

*• Metrics to measure radiologists’ impacts on patient outcomes are key.*

*• The value provided by radiology is multifaceted, going beyond exam volumes.*

## The concept

European governments, like those in other parts of the world, are increasingly facing difficulties in managing their national health systems. This is due to a variety of causes: the most important being an ageing population, a rise in the prevalence of chronic conditions and the accelerating pace of medical innovation—all of which have increased demand for state-of-the-art treatment. These factors, associated with a long-lasting economic crisis, constitute a severe threat towards maintaining and safeguarding the current levels of healthcare [1].

Short-term cost-cutting solutions and austerity measures have been the first reactions to difficulties. These have, however, already reached their limit and are now negatively affecting the quality of healthcare. At present, cost-saving and austerity are fuelling a paradoxical effect, creating a vicious circle of increased demands on healthcare and the need for greater spending.

Thus, there is a need to re-imagine how health services are financed.

The concept of value-based healthcare (VBH) has emerged as a framework for achieving better results, considering first those factors that matter most to patients, while optimising, at the same time, the cost of care delivery within the health system. Value is defined as health outcomes achieved for patients relative to the associated costs. Value (in this context) depends on the results of care and is measured by reference to the results obtained, and not to the volume of services delivered [2].

Within this concept, payments are assigned according to the outcomes of a given episode of care, and good outcomes have to be obtained in the most efficient way to achieve a reduction in associated costs.

At present, care to a patient (or a patient population) with any medical condition is usually delivered by a multitude of specialists, working in different units and departments, each taking care of the patient from the point of view of his/her expertise. In a value-based healthcare environment, the whole organisation has to change, and care for any medical condition is given by specifically dedicated multidisciplinary practice units with deep knowledge, a broad skill range and excellent facilities, which provide the full continuum of care to the patient. Such a model allows, on one hand, the development of the expertise necessary to achieve better short-term and long-term outcomes and, on the other hand allows the measurement and optimisation of the costs involved in the whole cycle of care. For acute conditions, these practices will mainly affect physicians working in hospitals. Chronic diseases, however, will see the involvement of a network of general practitioners (GPs) and specialists working outside of the hospital to ensure longitudinal follow-up of patients and evaluation of the final outcome of the care provided. Such changes are quite complex and require a “revolution” of the whole healthcare system, from an organisation centred on medical specialties to one centred on the patient and his/her needs. Furthermore, a profound rethinking of the role of hospitals in relation to their area of influence is needed, with the development of an active role for care management and improvement not only within the hospital itself but also for other providers in their network [3, 4].

Value measurements are based on a three-tiered framework (Table 1). Each tier contains two levels, each with one or more dimensions related to the state of health reached by the patient during and after treatment. The lowest tier (tier 1) measures “sustainability of health” (with “care-induced illnesses” and “recurrences” as internal dimensions); tier 2 relates to the “process of recovery” (internal dimensions are “diagnostic or treatment mistakes and their complications”, as well as “time to return to normal activities”); tier 3 is defined as “health status achieved or retained” (and its internal dimensions are “degree of health recovery” and “overall survival”) [1].

**Table 1:** Measuring value-based care

Tier 1	Sustainability of health	Care-induced illnesses Recurrences
Tier 2	Process of recovery	Diagnostic or treatment mistakes and their complications Time to return to normal activities
Tier 3	Health status achieved or retained	Degree of health recovery Overall survival

Measurements of both outcomes and costs are not easy. Teams of experts, including physicians, health economy experts and representatives from patients’ organisations, are working to identify metrics that allow measurement of the “significant outcomes” within each of the steps of the value frame [5]. The International Consortium for Health Outcome Measurements (ICHOM) has already published a series of 20 outcome measurement sets for 20 different clinical situations (such as breast, colon and lung cancer, stroke, dementia, coronary heart disease, and others). Other sets are under preparation. The organisation aims to publish standard sets to cover more than 50% of the global disease burden by the end of 2017 [6].

Simultaneously, health economists are working on the measurement of the costs of each step of the process, comprising materials, time, professional fees, etc. [7].

This may all seem sensible, but the value-based healthcare concept, as presently structured, is fundamentally flawed, not by what it seeks to achieve, but by what it does not even consider. Significantly, no radiologist has been involved in any of this work, and radiology is not even considered in the value-based healthcare concept. The framework used to measure health outcomes, in fact, starts only at the beginning of treatment (after most of the work of the radiologist has already been done). The entire diagnostic process, as a whole, is only considered as affecting tier 2, in case of errors or complications, and then is only considered as having a negative effect on outcomes; that is, when something goes wrong. Thus, a correct diagnosis seems to be taken for granted, as if it were a commodity, and as if the work of a radiologist was analogous to that of a machine for measuring blood biochemistry.

Radiologists play a fundamental role in the diagnostic process of modern healthcare delivery. However, they are often considered as factories producing imaging examinations, with attention focused only on the number of procedures performed. Their work is considered as a chain of processes and their results, the diagnoses, are not regarded as an outcome.

In the clinical projects implementing value-driven programmes that have been developed, radiology has been simply considered as a cost and measured as such. The diagnosis, and how it has been possible to reach it, has not been regarded as the first important result of an entire episode of care [8].

A patient, however, is not a disease or a pathological condition, for which it is possible to classify and measure the results of treatment. He/she is an individual who seeks help for signs and symptoms with a view to understanding and curing the underlying reason for them. A correct, timely and useful diagnosis is the first and crucially important step that matters to each patient and to other healthcare professionals.

Therefore, the establishment of a correct diagnosis is the first outcome that must be considered in the healthcare process. It is an intermediate outcome, but definitely an outcome nonetheless, and as such has to be considered in any value-based healthcare paradigm. In addition, the contribution of radiology to the exclusion of possible disease should be remembered; substantial benefit to patient outcomes can accrue from radiology investigations that exclude significant disease. In the present value-based healthcare model, none of these outcomes are measured, or even taken into consideration.

The topic of value-based radiology (VBR) has been addressed by many recent papers, most of them reflecting the ongoing debate within radiological societies and organisations in the United States. The authors have emphasised the importance of the active role that radiologists need to have in the transition from volume-based to value-based healthcare and have proposed new metrics that show the benefits provided by radiology to patients [9–14].

The situation in Europe is different from that in the United States. In many European countries, healthcare organisation relies on a national health system that acts as facilitator, organiser and payer, and provides care to citizens. In addition, the situation is not homogeneous throughout Europe, since the national systems differ in terms of organisation, governance and means of funding and payment. The concept of value-based healthcare, however, is being discussed also in Europe, and a few experimental implementations of the system have already been initiated. Therefore, it is necessary to understand the role of radiology in this new framework also from a European perspective, and to ensure that radiology is properly represented in the development of concepts and healthcare planning.

The European Society of Radiology (ESR) has established a working group on VBR. Its goals are:

- To develop a definition and conceptual framework for VBR in Europe and to embed value-based radiology as a strategic paradigm for the Society’s activities
- To ensure the ESR’s ability to respond, shape and manage healthcare trends towards value-based approaches relevant for medical imaging
- To increase and demonstrate the value radiology, radiology professionals and the ESR provide, and to improve cooperation with all relevant stakeholders
- To establish a strategy for enhancing the visibility and reputation of radiology, and for positioning the radiology profession within the healthcare sector vis-à-vis other medical professionals, patients, industry, political stakeholders and society at large
- To further develop the ESR’s programmes and projects in accordance with the concept of value-based radiology

The group is led by the current ESR first Vice-President and initially comprised the Chair of the ESR Board of Directors, the ESR Past-President, the Quality, Safety and Standards Committee Chair, the Education Committee Chair, the National Societies Committee Chair, the PIER Subcommittee Chair and the EuroSafe Imaging Chair and industry liaison. Three group meetings were held in 2016 and in 2017, and the ESR Patients Advisory Group joined the working group in May 2017.

Our working group is facing a daunting task. It is difficult to precisely define the concept of value-based radiology, and it is even more problematic to develop metrics which would allow a clear demonstration of the benefits contributed by imaging to the care of patients.

There are many reasons for this. The most important is that radiologists’ work is not performed in isolation. Our results depend both on the appropriateness of referrals and on how our reports are understood and used by the physicians who treat the patients. Even the outcomes of the therapeutic procedures performed by interventional radiologists are linked to those of the other doctors who precede and follow the intervention. In emergencies such as trauma and stroke it is even more complicated, since the time from an accident or from the beginning of symptoms to diagnosis and treatment are critical factors, which depend on the organisation of the whole emergency system, not on that of the hospital and radiology department alone. An altogether other issue is the problem of measuring the costs of radiation protection measures (which are enforced by the 2013/59/Euratom Directive in Europe) [15], the difficulty in fully assessing patients’ experiences throughout the diagnostic process, and the difficulties in understanding how continuous professional education, teaching and research activities impact on the final diagnosis of each patient.

## The metrics

The activities chain within the radiology department, commonly called the “value chain”, has been explored in a number of papers [14, 16, 17]. Each of the steps in the value chain is composed of many different processes, and radiologists are quite good at measuring and improving these. But improving one or more processes does not necessarily lead to better outcomes.

Although most potential improvements of certain steps aim at workflow efficiency optimisation only, some are directed at aspects of the activities chain that are quite important for a high-quality diagnosis (our “outcome”).

We believe that five of these process steps can be regarded as key factors:

1. Appropriateness of requests
2. Attention to radiation protection measures
3. Characteristics of the reports (correct, complete, well understandable, structured and properly used)
4. Relationships between patients and radiology personnel
5. Continuous professional education, research and innovation

These steps address the most important ethical “values” in our discipline: attention to patients’ safety and wellbeing, attention to the quality of all aspects of the work, and development of good relationships with patients and referring physicians. All are considered as important not only by radiologists but also by the patients who benefit from our services [18]. The development of metrics that allow evaluation of these parameters would possibly facilitate some measuring of the quality of the diagnostic outcome. Together with an assessment of the costs involved, a measure of the final “value” of radiology could be obtained.

We should not forget that metrics are a surrogate for “truth”, and not the actual care provided to and experienced by patients and their families. Furthermore, they can be quite complex and difficult to measure accurately; some can address the same point from different angles and some may deal with intangible assets whose direct relation to outcome can be hard to demonstrate.

### Appropriateness of requests

This first step in the activity chain does not depend on radiologists alone. Although it would be desirable to have a discussion between the referring physician and radiologist about the most-appropriate imaging test to evaluate each patient, the workload is too great and this is not feasible in clinical practice. Personal contact before the examination is usually sought in a few, quite difficult cases only. Appropriateness, however, together with awareness of radiation risk and audit, are at the foundation of the justification process for every planned study. Provision of the right exam to the right patient, at the right moment and for the right clinical indication, is of the greatest importance, and, in terms of defining value, is one of the most important contributions of radiologists to the wellbeing of patients.

Appropriateness can be measured through:

1. Analysis of compliance of requests by referring physicians with (institutionally approved) imaging referral guidelines
2. Identification of duplicate studies
3. Rejection of unnecessary or redundant studies
4. Time and frequency of referring physician consultation
5. Ease of availability of radiologists for referrer consultation [15]

The radiological community, both in Europe and in other countries, has developed clinical decision support (CDS) systems to ensure a consistent and appropriate utilisation of resources by referring physicians. Their use would easily allow measurement of these metrics [19]. In Europe, in order to harmonise imaging appropriateness criteria throughout the different countries, the ESR has recently launched a computer-based CDS tool called ESR iGuide. This provides a core standard system, with guidelines adaptable to local (national and institutional) situations [20], and its implementation and clinical use have already started in different countries.

### Radiation protection measures

The 2013/59/Euratom Directive enforces, among other things, the use of medical radiation protection measures in Europe. Although it would be quite difficult to calculate the real benefit of the use of such measures on the quality of our principal outcome, the final diagnosis, radiologists must pay attention to this topic by choosing a non-ionising examination technique whenever possible and the optimisation of procedures. Furthermore, the Directive strengthens and expands the previous requirements regarding diagnostic reference levels and reinforces the need for education in this field of all involved personnel and of medical students [21]. A number of metrics concerning radiation protection can be put in place:

1. Presence of diagnostic protocols which entail choice of non-ionising examinations whenever possible
2. Presence of “low-dose” protocols in all computed tomography (CT) equipment
3. Percentage of use of such protocols
4. Reporting to a radiation dose index registry of all exposures
5. Evidence of training programmes on radiation protection aspects

### Characteristics of the radiology report

The report is an important aspect of our work, since it is the gateway of communication with other healthcare providers (and increasingly also with the patient). Ideally, the report should provide the final answer to the reason why the patient was referred. There is consensus in the literature about its final characteristics: a good report should be timely, correct, complete and “actionable” [12, 16]. Therefore, it has to be evaluated for completeness, accuracy, clarity, specificity, adherence to guidelines and disease-based structure. There is a strong trend to move away from “prose” reports towards “structured” reports, and to that effect templates are being developed. There are possible metrics on the quality of the report that can be established within radiology departments. They can be obtained, for instance, through measurement of the time from request to reporting, the number of discrepancies/errors meetings within a department (and, possibly, by the number of discrepancies/errors found) and the establishment and regular use of disease-specific structured reports that document information of unique importance for each condition. The latter data are a measure of accuracy and completeness.

However, how a radiology report is understood and used by referring physicians does not depend on radiologists alone (and this is probably the most important aspect, directly related to the final outcome of the episode of care). It is known that after direct consultation between radiologists and referrers, be it direct person-to-person contact or during structured clinical-radiological meetings [22, 23], significant new information can be obtained and that (especially in cancer patients) both major and minor changes in diagnostic and therapeutic management occur quite frequently. Thus, metrics about the impact of the report on patient management can be obtained through measurements of the number of formal meetings between radiologists and other specialists, the number of cases discussed in each of them and the percentage of cases in which significant changes in therapy are decided after direct consultation. In some countries, programmes have been developed to measure many of these quality and value parameters on a nationwide basis [24].

### Relationships between patients and radiology personnel

This topic does not relate to customer satisfaction only. It deals with the entire patient-doctor relationship and, in radiology, is directly related to our “visibility” to patients. Availability to talk before, during and after the examination is probably the best way to improve the quality of the “radiological experience” for each patient, and this can involve any of the personnel dealing with them, from clerks at admission to nurses, radiographers and radiologists. Any conversation, furthermore, should be polite and respectful. Availability is an “intangible asset” that may be difficult to measure properly, and assessing courtesy towards patients is even more difficult. Some metrics, however, can be established:

1. Presence within the department of detailed instructions for preparation for the different types of examinations and the percentage of patients receiving them
2. Distribution of customer satisfaction questionnaires (and these are better if they have been developed together with patients’ representative organisations) and periodic audits based on their results
3. Existence of formal relationships between the department and patients’ organisations

Other metrics may relate more closely to the direct patient-radiologist relationship.

Although it is not clear from the literature if patients prefer having the examination results directly communicated to them by the radiologist or by the referring physician [25, 26], a departmental policy that facilitates availability for queries and explanations, when requested, should be in place and measured. Agreement with patient organisations as to whether this should be a short in-person consultation upon request or specific time slots during the day would be preferable.

Furthermore, other methods of delivering examination results to patients, such as online portals or patient gateways, through which patients can directly access their own results, may also be considered.

### Continuous professional education, research and innovation

This is probably the most difficult topic for which to identify metrics. It is clear that the ability to learn, improve and innovate is directly related to the quality of our final outcome, the diagnosis. In any company, it is only through the ability to launch new products, create more value for customers and continually improve operating efficiencies that new markets can be reached, increased revenues and margins obtained and value for stakeholders created [27, 28]. However, improvement through education and innovation takes time to impact results, and often does so indirectly. It is, therefore, difficult to understand how to measure its effects. This is even more complicated in the medical sciences, where transition from theoretical knowledge into clinical practice may take a long time and may not be straightforward.

Continuous medical education (CME) throughout one’s whole career is a duty of all European doctors, radiologists included. There are some differences in the rules established by the regulatory bodies of each country, but the same general principles apply throughout Europe [29, 30]. Compliance with national regulations regarding CME can be used as a metric, and the number of CME points collected per year by each radiologist can be an additional one.

In recent decades, the growing complexity of our discipline and the need to provide high-quality services have led to the evolution of a number of recognised radiological subspecialties. A relatively large number of radiologists nowadays devote their practice to one or more subspecialty area(s), and this is true especially in large hospitals and academic centres. Deeper knowledge of the clinical and radiological aspects of the chosen topics and capability to have a better dialogue with the respective clinical specialists are well-known advantages of subspecialisation in radiology. Furthermore, it has been shown that second opinions obtained from consultations with subspecialised radiologists may result in clinically important differences in detection and interpretation, in comparison to the reports issued by a general radiologist. Thus, the number of examinations read by subspecialists (either as a primary reading or as a secondary consultation) can be used as a metric relating to the quality of the radiology report [31–35]. Such services, however, are not available on a 24-h basis, and it is difficult to organise departmental activities to ensure that the best possible competence can be offered to all patients at all times [36]. Both the presence of an organisational structure that allows re-evaluation of out-of-hour studies from subspecialists the next working day, or in which it is possible to have on-call consultation from subspecialists, as well as the usage rate of such services, can therefore be used as additional metrics.

Things get even more complicated when it comes to research and innovation, since the translation of new discoveries from bench to bedside is usually quite slow, and it is not possible to measure the impact of research productivity of a radiologist, or of the whole radiological department, on the final diagnosis of each patient. However, there is no doubt that research is the future of any clinical discipline, including radiology, and being active in research is key to keeping up to date with clinical and technical advances in our discipline. Therefore, at least in theory, research productivity within a department can be linked to the quality of the medical services provided. Metrics about this topic can include the number and quality of papers published, the number of patents, and the amount of research funding received per year.

### Interventional radiology

Interventional radiology is somewhat different from diagnostic imaging, since the results of interventional procedures can be considered directly as outcomes under existing models. They fit perfectly within the value-based healthcare framework: patients’ preferences can be assessed, costs can be measured and “value” calculated and compared to that of other therapeutic procedures. However, interventional radiology procedures are not performed in isolation. As already noted, their outcome is linked to the work of the other doctors who precede and follow the intervention and are thus subject to the quality of both the referral and follow-up. Furthermore, in the existing VBH framework, no “value” can be calculated regarding the correct choice, the quality and the results of the diagnostic examinations performed to decide on the feasibility of the procedure and to guide it, and these are often performed immediately before the intervention, in the same session, and often by the same radiologist. It is not only a matter of correct diagnosis; the details of how to perform the therapeutic procedure can often be chosen only from data obtained by specifically tailored diagnostic examinations. The quality of their results is therefore critical in determining the “value” of the following therapeutic procedure. These too have to be considered and metrics about them must be developed.

## The perspective

The concept of value-based healthcare has been developed as a response to the financial pressures that are causing crises in our health systems. Although driven, at least in part, by financial imperatives, value-based healthcare can be seen from two different angles: it represents not only a necessary new approach to guarantee financial sustainability but is also important in and of itself as a focal point for evidence-based, measurable and outcome-driven healthcare. This second aspect would be greatly significant, in fact, even in a system with unlimited resources. So there is both a *financial need* and a *compelling qualitative reason* to re-imagine health services; therefore, the current situation is also an opportunity to improve healthcare in this sense [37].

In the current thinking on value-based healthcare, we sadly note that the diagnostic process, as a whole, is not considered in the outcome evaluation process. Measurements start at the beginning of the therapeutic management of the patient, after the diagnosis has already been established. In the currently prevailing model, the work of diagnosticians (radiologists, pathologists, laboratory medicine specialists and even the family doctor to whom the patient first goes when health problems arise) is not considered.

The ESR has decided to take a three-step approach to this topic. The first is dedicated to determining a number of metrics about topics that both radiologists and patients believe are most important for the quality of our “outcome”. These are addressed in this article.

The second step involves the creation of a general assessment programme of the activities performed by radiologists. This should consider these metrics and take into account all the different perspectives from which the metrics can be evaluated (financial, radiological, patients’ and innovative). Hopefully, this approach will allow us to begin a discussion about the criteria by which radiology departments are assessed: not only on the number of examinations performed but also on the quality of their diagnostic outcome [37].

The third step (similar to what has been done for the outcome measurements of different diseases by ICHOM) relates to the identification of a number of different clinical situations in which it is possible to analyse the diagnostic process, delineate specific metrics along its course and measure the final quality of the outcome, taking into consideration both internal and external factors affecting it. Examples could be: in patients with stroke or trauma, the time from the accident or onset of symptoms to imaging and image-guided therapeutic procedures; or (this would be even more complicated) considering the integration of radiological findings with physical examination, history-taking, laboratory tests and even pathological results in subjects with complex conditions, to “measure” the results of the whole diagnostic process. Another example could be the influence of a radiological examination on subsequent patient pathways after referral to an emergency department (e.g. findings of an abdominal CT help to decide whether a patient will be transferred to gastroenterology or abdominal surgery).

This article reflects the results of the first discussions within the ESR Value-Based Imaging Working Group and attempts to indicate possible metrics useful to calculate all the quality aspects of the outcome of the activities of a radiology department: the diagnosis. Its aim is, first and foremost, to launch a discussion within the ESR, and the radiological community as a whole, about this topic. Comments and suggestions about the general concept of VBR and the metrics that have been tentatively suggested are needed in order to understand if these are considered as useful throughout the different European settings, to build consensus about them and to guide the ESR’s strategy.

The discussions on the VBR concept will therefore continue.

First of all, the Working Group needs to involve other key stakeholders. The ESR Patient Advisory Group has already discussed this topic and joined the Working Group at its May 2017 meeting. Representatives from associated industries also need to be involved. There are many challenges in the accounting and control systems in healthcare given the complexity of the core operating processes, in addition to the multiple and often conflicting sets of goals imposed on healthcare organisations by internal and external stakeholders and the highly politicised environment. The development of a new full set of metrics adds to the already existing difficulties and can be perceived as an additional severe burden imposed on radiology management. The technical feasibility and the ease of monitoring these new metrics have to be considered. Medical informatics solutions have to be found to facilitate measuring them easily, frequently and reliably.

However, to calculate the “value” of a diagnosis, all the metrics and the outcomes we have discussed will have to be related to the costs of achieving them. Therefore, it will be necessary to work on this aspect too.

Finally, any activity on the VBR concept needs to be shared and discussed with, as well as accepted by, external stakeholders. It will be, first of all, necessary to have the concept that a radiological diagnosis is a definite “outcome” accepted by administrators, health economists, payers and political authorities, so that the contribution of radiology is considered as a valuable component in any future value-based healthcare model.

The discussion has just started…
